# Impact of climate risk materialization and ecological deterioration on house prices in Mar Menor, Spain

**DOI:** 10.1038/s41598-023-39022-8

**Published:** 2023-07-21

**Authors:** Matías Lamas Rodríguez, Mari Luz Garcia Lorenzo, Manuel Medina Magro, Gabriel Perez Quiros

**Affiliations:** 1 Banco de España, Madrid, Spain; 2 Universidad Complutense de Madrid, Madrid, Spain; 3 Universidad of Alicante, Alicante, Spain; 4 CEPR, London, UK

**Keywords:** Environmental impact, Environmental economics

## Abstract

The frequency and severity of extreme events related to climate change have intensified worldwide in the last decades. It is documented that increasing extreme rainfall and flooding cause more nutrient runoff into waterbodies, initiating numerous harmful algal bloom (HAB) events, especially in fragile ecosystems. We analyze the dramatic economic damage of one of these episodes in Mar Menor, the largest salt-water lagoon in Europe. We show that when the public perceived the severity of environmental degradation, the return on housing investment was 43% lower in the surroundings than in similar neighboring zones 6 years after the HAB (2015). This represents a loss in housing wealth of more than 4000 million euros, around ten times the gains of changing from dry-farming to irrigated crops, which makes this ecosystem fragile. Hence, we quantify some of the economic consequences of ecological deterioration linked to episodes of Global Climate Change.

## Introduction

Coastal lagoons, as stated by the Habitats Directive of the European Union^1^, are designated as endangered areas that require immediate environmental protection. These areas and their surroundings are often under high anthropogenic pressures and undergo significant socio-economic and environmental changes over the years, being an example of the conflict of interests between the development of human activities and the ecological requirements of the aquatic system^2^. Moreover, coastal lagoons are among the most threatened marine ecosystems due to human pressures in the catchment areas, which often result in the release of nutrients that may lead to eutrophication. The effects of eutrophication are further amplified by global climate change^3–5^.

**Figure 1:**
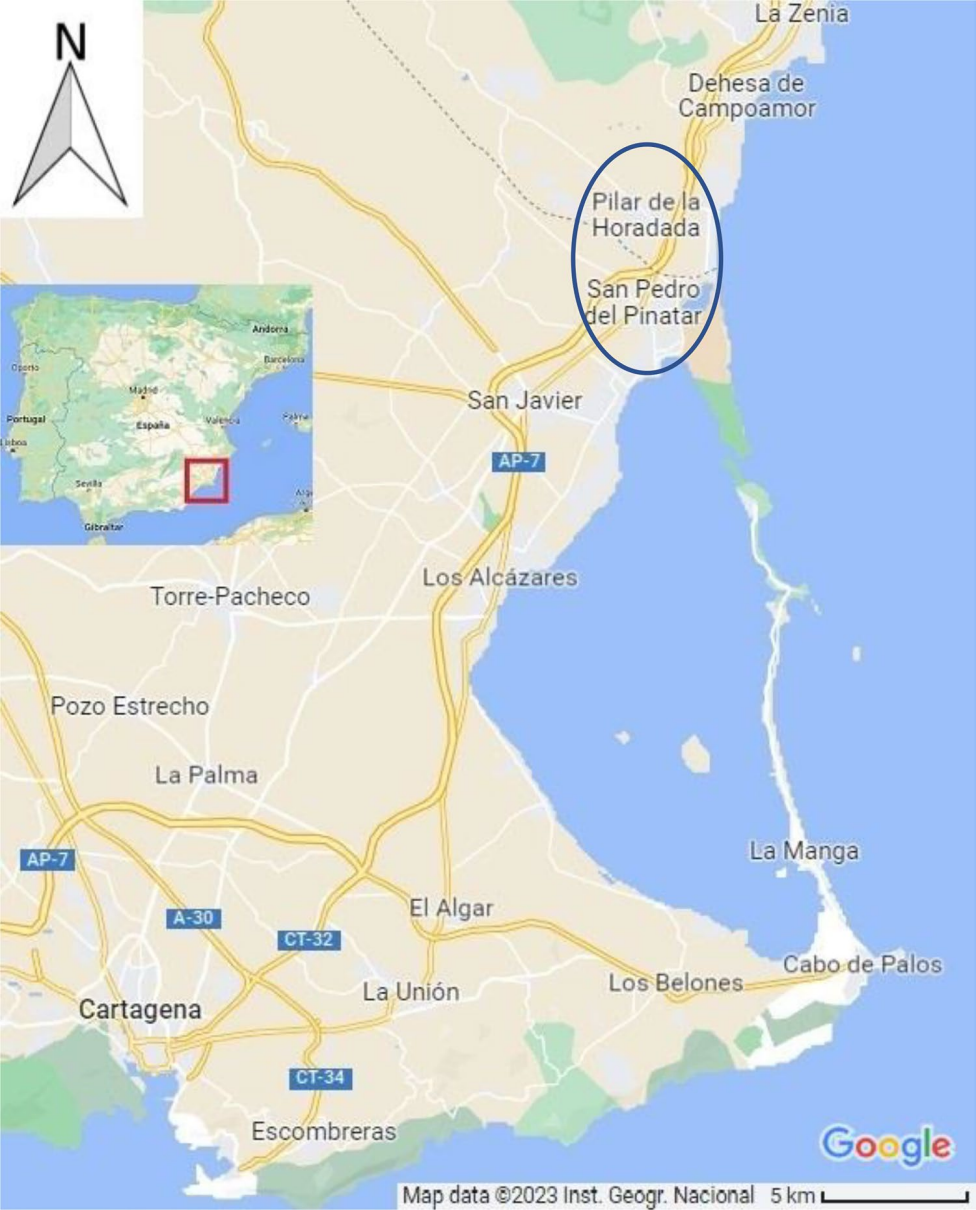
Mar Menor. Map and Location. The blue oval shows San Pedro del Pinatar, the northernmost municipality of Murcia on the shore of the Mar Menor, and Pilar de la Horadada, the southernmost municipality of Alicante on the shore of the Mediterranean Sea, both analyzed in a later section. Source: Google Maps. Retrieved 12 June 12th 2023. https://www.google.com/maps/@37.7162418,-0.9252364,11z?entry=ttu.

Nutrient enrichment is known to stimulate phytoplankton productivity, and the continuous accumulation of organic matter can lead to the eutrophication of the system with a series of damaging ecological effects that can also be harmful to humans^6^. Harmful algal blooms (HABs) are often associated with the eutrophication of coastal waters and estuaries. This rapid increase in the population of algae has detrimental effects on aquatic life triggered by hypoxic waters. An example of this phenomenon recently occurred in the Mar Menor, a coastal lagoon in the southeast of  Spain (Fig. 1). This lagoon has undergone significant environmental degradation in recent years, primarily due to excessive external nutrient input, especially from non-point source pollution^2^. As a result of this degradation, the Mar Menor recently suffered various notable episodes of HAB.

The main pressures on the Mar Menor are agriculture and tourism^7,8^. The urban-tourist development in the Mar Menor region began in the 1960s and primarily focused on the coastline. As a result, one of the first pressure was the discharge of urban wastewater that historically affected the water quality of the lagoon, particularly during the summer months, due to the overloading of the treatment infrastructures^9^.

Simultaneously, the tourism sector has demanded the development of various infrastructures, including roads, ports, dikes, boardwalks, and beach regeneration projects. However, these constructions often come with accompanying environmental impacts. For instance, the opening of the Estacio canal in 1973, which improved water connectivity between the lagoon and the Mediterranean Sea, caused a reduction in the lagoon's salinity and affected its temperature range, leading to significant changes in the lagoon’s ecosystems and dynamics^9^.

However, the main cause of degradation in the Mar Menor ecosystem stems from the uncontrolled expansion of the agricultural sector and agri-food industry throughout the watershed, especially since the conversion from dry-farming to irrigated agriculture (nowadays, more than 80% of the total area)^7–9^.

Irrigation in the Mar Menor region is maintained through a combination of resources, including water from the Tagus-Segura aqueduct, desalinated seawater, reused wastewater, and groundwater sourced from the Quaternary aquifer. The use of water from saline aquifers requires a desalination process, which generates brine rich in nitrates^10^.

Estimates indicate that agricultural activity is responsible for around 85% of the total nutrient input into the lagoon, while urban activities contribute to the remaining 15%^9–11^. This nutrient input has led to severe eutrophication and water quality deterioration in the lagoon. Additionally, intensive agriculture has resulted in nitrate contamination of aquifers, exceeding the acceptable limits. All of these increasing nutrient concentrations lead the lagoon to eutrophication, subsequently generating the HAB and jellyfish proliferation^5^.

Mar Menor water quality and benthic communities were detected to have deteriorated during the summer of 2015. Deterioration reached alarming levels by early 2016^12,13^. A recent report by the Spanish Institute of Oceanography on the evolution and current state of Mar Menor^14^ corroborates that the end of 2015 is a turning point in its ecological evolution. According to this report, the analysis of 50 years of time series data shows that the chlorophyll levels in the lagoon stood within a range characteristic of a low nutrient system until the first phytoplankton proliferation episode in the summer of 2015.

Additionally, HAB episodes in Mar Menor have been frequently associated with floods or extreme temperatures in the region (9 episodes of flooding events with relevant or catastrophic damage have been documented in the period 2000–2022, 5 in the last 6 years, versus 5 in the previous 50 years)^15^. This frequency of extreme episodes is widely documented to be related with climate change, all over the World^16,17^ and specially on the Mediterranean coast of Spain^18–20^. The increasing extreme rainfall and flooding have been reported to play an important role in the dynamic changes of nitrogen and phosphorus nutrient cycles, promoting eutrophication and the expansion of algal blooms^21,22^.

This paper shows what happens when climate risk becomes a reality affecting a fragile ecosystem. Our analysis suggests that, in line with the literature on climate risk finance^23,24^, assets in Mar Menor did not take into account the possibility and the consequences of climate related risks on the lagoon. It is only when these risks materialize and when the public becomes aware of them, that climate-sensitive assets, such as real estate, adjust their prices accordingly. Albeit we focus on a local phenomenon, HABs in Mar Menor and their impact on house prices in this area, our results are indicative of the potential for a broad mispricing of assets subject to climate-related risk, which might be more prominent in similar areas subject to human pressures. This viewpoint, and the effects of this mispricing of assets on financial stability, is clearly stated in different official documents from all major central banks and international organizations^25–30^.

As we will see in the next section, the economic impact of HABs in different areas, but also in Mar Menor, has been assessed in different papers. However, the existing literature tend to focus on measuring partial costs (e.g., costs to specific sectors in the economy), which are likely to vastly underestimate the total costs stemming from environmental degradation. To overcome this limitation, we will take a catch-all measure of these costs by considering house prices in the Mar Menor. House prices are appropriate for this goal because, as we will elaborate later on, they have been shown to be a valid proxy for the overall quality of life and economic conditions in a region.

## Results

### Economic effects of the HAB

The economic consequences of HAB episodes have been widely documented^31^. The literature describes four different aspects: human health impacts^32,33^ (health care costs, and loss in productivity because of work absenteeism); commercial fishery impacts^34,35^ (fish mortality due to oxygen depletion and decrease in consumer demand due to potential contamination); tourism, leisure industry and recreation impacts^36,37^ (decrease in hotel reservations, restaurant bookings and other recreational activities); and, monitoring, management and restoration impacts^38,39^ (costs of cleaning the water or controlling its quality).

However, even when the documented costs are high, it only gets to the magnitude of billions when describing, not an individual case, but, for example, all the cases in the US^40^. We will show in this work that the measurement of partial costs, only for specific sectors, sharply underestimates the total costs. This bias has serious consequences because responses to HABs have been analyzed, formulated, and implemented with a partial and inadequate understanding of the net benefits of such responses^41^.

Mar Menor is a good case study of the underestimation of damage caused by environmental degradation. Several studies have attempted to measure the economic cost of this environmental catastrophe. One approach has been to compare Social Security registrations in Mar Menor municipalities with those of neighboring municipalities, but this approach fails to obtain robust or statistically significant findings^42^. A very recent approach calculates the probability of a business failure in the Mar Menor surroundings associated with an increase in the concentration of chlorophyll, showing clearly that there are winners and losers by sector, with a decrease in the probability of failure in the Agricultural and Transport sectors, but a substantial increase in Industry and Building, Financial and Real Estate, Major and Minor trade, and Accommodation^43^. Another approach uses surveys to measure the willingness to pay for clean waters, but the obtained values are too low to support any cost–benefit analysis of restoration policies^44^. Other researchers conduct surveys on residents and visitors to identify stakeholder perception of the environment and to ascertain how much they value the importance of ecological processes in order to maintain the ecosystem^45^. These surveys also appear to underestimate the economic consequences of the Mar Menor’s degradation, since they fail to align adequately with the growing societal debate contained in the press and reflected in social protests.

### HAB and house prices

In this article we step away from measuring what people subjectively claim to be the impact of HAB episodes and from measuring the evolution of some specific sector of the economy. We quantify what people are actually paying to enjoy the Mar Menor’s ecosystem. If walking down the shore of Mar Menor is less enjoyable than it was before, if there are fewer bars and restaurants, or if the fishing activities are less productive, not to mention the health impacts, people may be less willing to live there and or rent/buy a holiday house there^43^. Against this backdrop, house prices provide a good indication of the quality of life in a neighborhood as has been shown in the theoretical and empirical economic literature^46,47^. Therefore, the overall economic impact brought in by HAB will be reflected in the Mar Menor house prices. In turn, we could use house prices as a shadow price that consumers (residents and visitors) assign to the evolving –and deteriorating- natural environment provided by the Mar Menor.

We are not the first researchers to analyze the effects of environmental degradation on house prices. There is a substantial literature on air pollution and house prices that, in general, suggests that house prices do not respond as much as expected to pollution—given the effects of pollution on health-^48^ although the disparity is high across studies^49^. This could be due to the difficulty of measuring future pollution in a given location, lack of knowledge of the effects of the different pollutants on health, and certainly the difficulty of accounting for all the other determinants of house prices. There is also a growing literature showing the effect of HAB on housing prices^50,51^. We note, however, that this latter research has mainly focused on low populated areas or on HABs that were short-lived. This is different from the case of the Mar Menor where HABs have received considerable attention from the public because, among other aspects, the Mar Menor is a densely populated area and a popular tourist destination in Spain. Therefore, it is not a surprise that house prices have exhibited greater sensitiveness to the HABs in our case study.

### Public perception of the HAB

HAB in Mar Menor has been perceived by the general public because it has resulted in a change in the color of the water (“the green soup”^52^) and, on several occasions, has led to large amounts of dead fish washing up on the shore. Even though the HAB is a continuous phenomenon and the public has been alerted on several occasions by different environmental organizations and academic experts since the mid-90s^53,54^, we will show in this paper that, only once there is visible material impact, people and societies do react.

**Figure 2:**
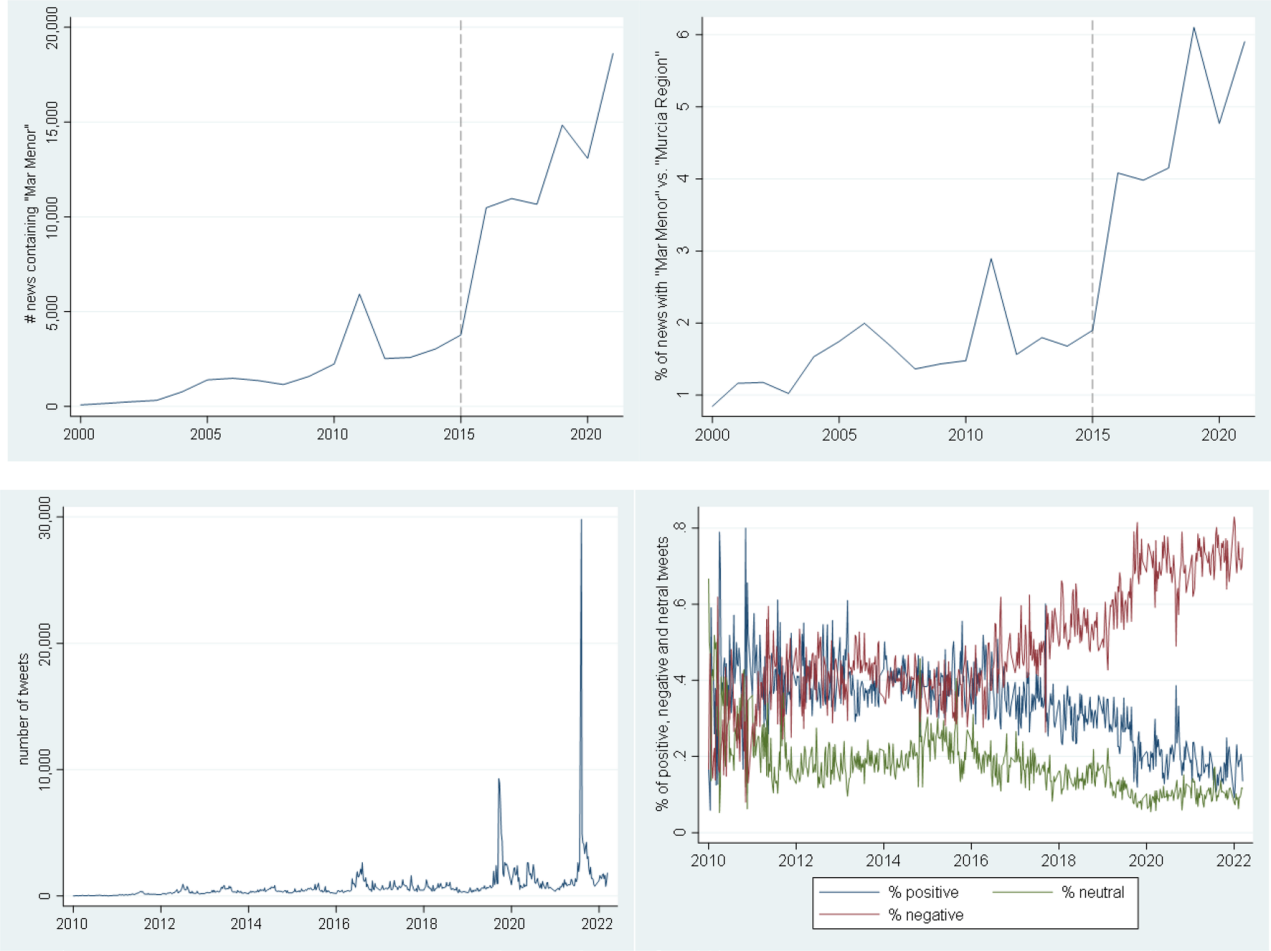
Public perception of the HAB in Mar Menor. Top left: Total number of news containing “Mar Menor”. Top right: Proportion of news with “Mar Menor” over news about “Murcia Region” (in %). Bottom left: Total number of tweets with the word “Mar Menor”, Bottom right: Proportion of positive, negative and neutral tweets with respect to the total number of tweets. Source: Factiva (Dow Jones Company) and Twitter.

Using Factiva, a business information and research tool which aggregates more than 32,000 news sources, commonly used to measure sentiment^55,56^, we can observe that this social concern was reflected in the number of news with the word “Mar Menor” (Fig. 2 top left), even when controlling for the number of news related to Murcia Region and excluding sports (Fig. 2, top right).

As can be seen in Fig. 2, and it is statistically tested^57^ (Supplement 1.1), the year 2015 constitutes a break point in the dynamics of the news series. Besides, the different peaks that can be seen in the figure (top panels) are clearly associated with significant moments. The peak in 2011 was an episode of a jellyfish plague, a typical precursor of a HAB; October 2016 is the first massive change in the color of the water; October 2019 is the first episode of dead fish on the coastal area; and finally, August 2021 is the last episode of dead fish. Additionally, the news by subject change dramatically in 2015 (from leisure to politics, Supplement 1.2).

But the public’s perception of the situation is not only reflected in the news. Figure 2 (bottom left) shows the number of tweets which included “Mar Menor”. As shown, the weekly number of tweets mentioning “Mar Menor” increased from 1022 to 9570 during the first episode of death fish on the shore, and from 2033 to 31,082 during the second. Figure 2 (bottom right) is even more revealing. It presents the proportion of positive, neutral and negative tweets that contain the words “Mar Menor”. These tweets have been filtered using machine learning algorithms and rule-based models^58,59^ (Supplement 2.1 provides details on the filtering). The test for structural breaks in the dynamics of the tweets also shows statistically different dynamics starting in 2016 (Supplement 2.2).

### A tale of two cities

After showing how the public perception of the Mar Menor has changed, and before discussing the estimation procedure, we set the scene with a brief tale of two cities adjacent to the area under analysis.

**Figure 3:**
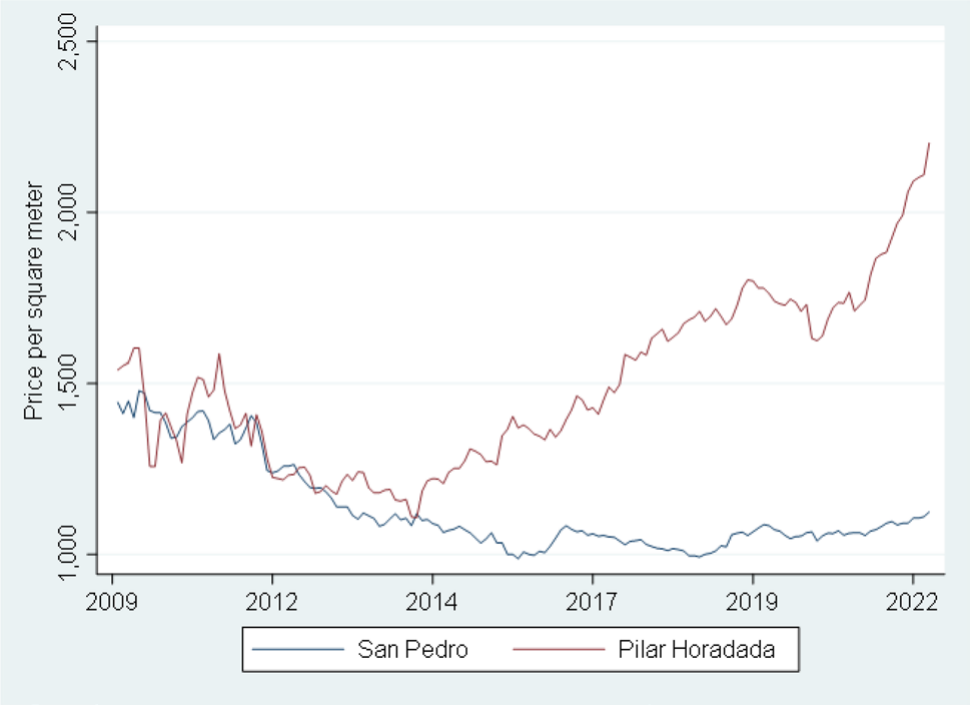
Monthly price per square meter in Pilar de Horadada municipality (control area) and San Pedro del Pinatar (affected districts). Source: www.idealista.com.

Figure 3 plots the price per square meter in two cities. Pilar de la Horadada (not affected by HAB) and San Pedro del Pinatar (Mar Menor) (Fig. 1 blue circle provides the exact location). These two municipalities are adjacent. The data comes from the most popular Spanish web page where owners put their houses up for sale (idealista.com).

A simple examination of the plot reveals that prices in the HAB area have failed to keep up pace with prices in the unaffected zone. We confirm this result by formally testing for cointegration^60^ in the log of prices of the two series. Cointegration implies that there is a long-term relationship among the variables and if they are separated in the short-run, they have the tendency to come back together. We run cointegration tests^61^ for two different samples, one ending in the summer of 2015 and another one for the full sample. In the first sample we find cointegration (there is a long-term relationship between these two series of prices). In the second sample we find no cointegration (there is no long term relationship). We formally test that the perception of the ecological degradation, measured as the difference in the proportion of positive and negative tweets, explains the gap between these two price series since 2015 (see Supplement 3 for details of the cointegration analysis).

### Data characteristics: treatment vs control areas

Even though the previous illustration was appealing, the prices analyzed before were ask prices, not transaction prices. Besides, prices on the real estate intermediary webpage might not be representative of the universe of transactions, and lack granularity (details on individual house unit characteristics). In the rest of this article, we propose a more comprehensive methodology to account for the previous issues, and to estimate more precisely the price effects of the HAB. To that end, we exploit data from the Association of Registrars, whose databases cover the universe of housing transactions in Spain at the individual property level.

**Figure 4:**
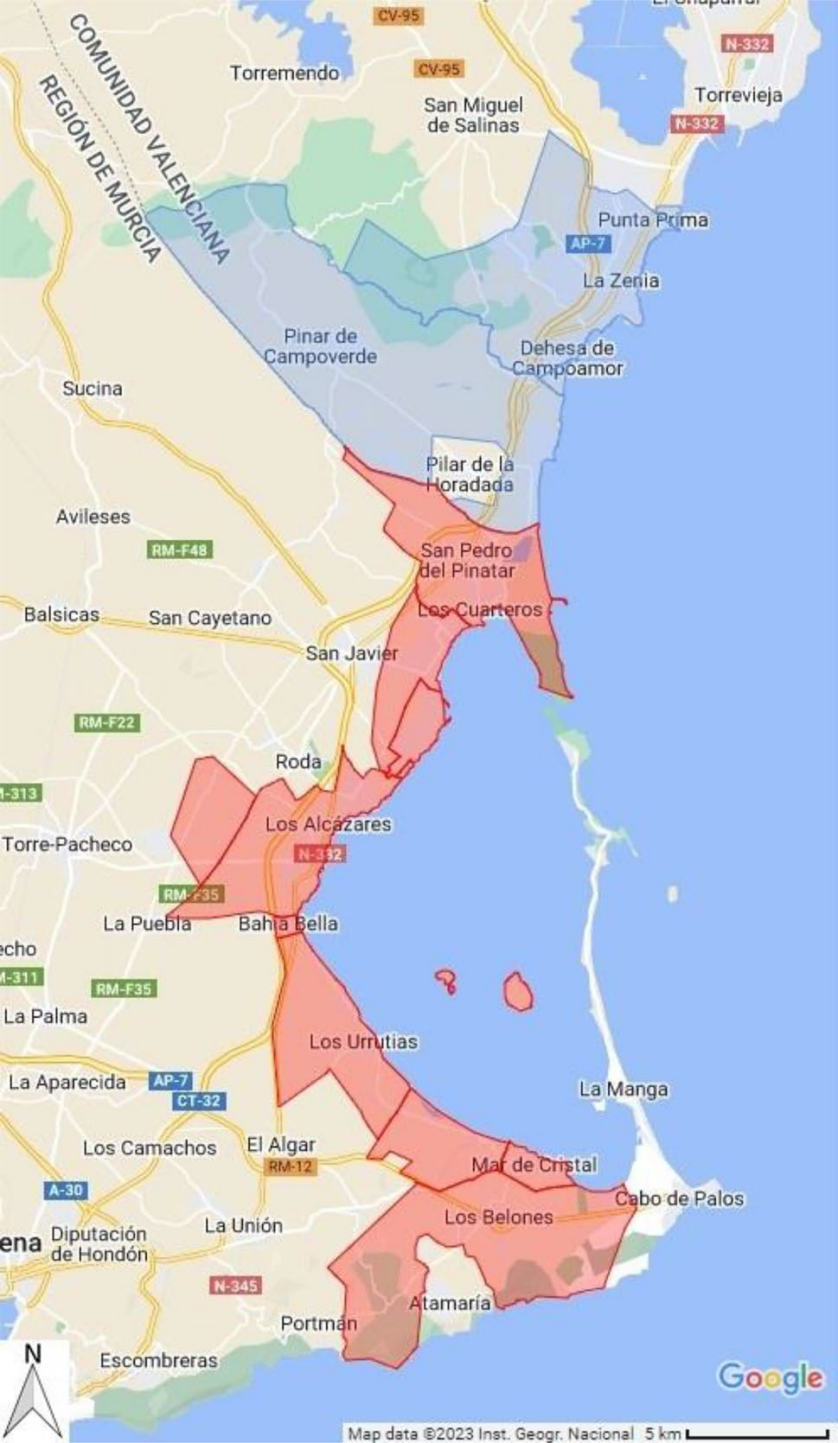
Red area represents the postal codes affected by the ecological deterioration, Mar Menor, approximately 164 km^2^. Blue area is the control group, the postal codes of south Alicante, approximately 91.4 km^2^. Polygons were drawn with gmplot 1.4.1 (Python library: https://pypi.org/project/gmplot/) using data for ZIP coordinates from Goerlich^62^ that processes data from Centro Nacional de Información Geográfica (CNIG). Work derived from: “CartoCiudad 2006–2021 CC-BY 4.0 scne.es” and “BDLJE CC-BY 4.0 ign.es”. Source of map: Google Maps.

The variable analyzed is the price per square meter of housing sold in the areas of interest. Dwellings are grouped by postcodes since 2013^62^, enabling us to distinguish between the areas affected by the ecological deterioration (Mar Menor, zones in red in Fig. 4) from similar but unaffected coastal areas (South Alicante, zones in blue). This control area borders to the north of Mar Menor and is made up of similar dwellings as we will show later on. To the south, Mar Menor adjoins Cartagena, a city area with a big port and very different characteristics from the coastal zone that concerns us (and is thus not included in the control group of dwellings). The number of transactions in this 8-year period is 13,260 in the control area and 8842 in Mar Menor. From Mar Menor we exclude La Manga, because, among other reasons^63^, it is only partially affected by the HAB.

### Returns on investment in Real Estate in treatment and control areas

**Figure 5:**
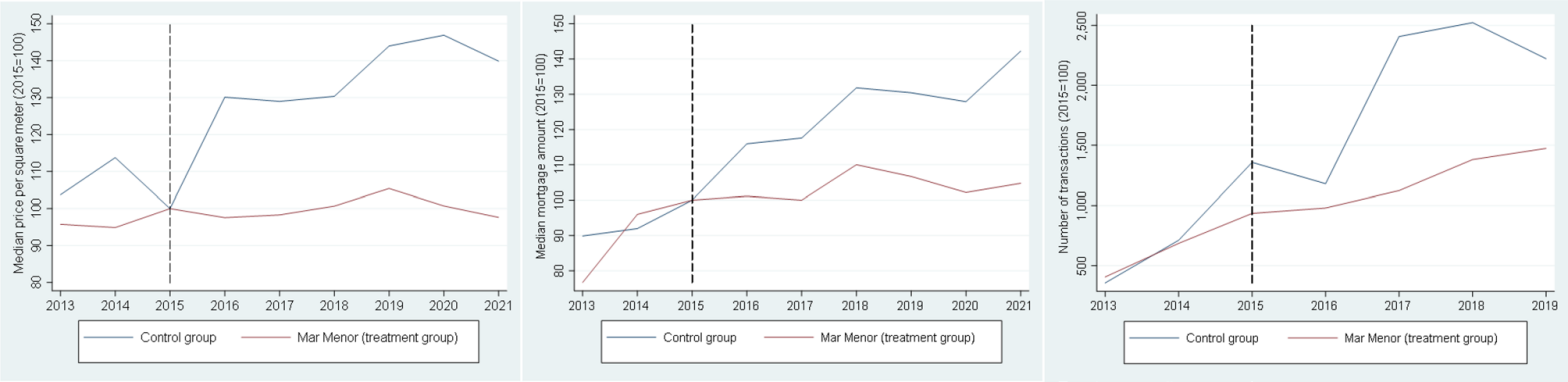
Price per square meter (2015 = 100) in the control area and Mar Menor (left). Median mortgage amount (2015 = 100) in the control area and Mar Menor (middle). Total number of transactions in the control area and Mar Menor (right). Last graph ends in 2019 to avoid the distortion in transactions associated with the Covid crisis.

Figure 5 (left) shows the evolution of the median price per square meter in these two areas. The graph is an index normalized to 2015 = 100. As can be appreciated, in Mar Menor the median return of an investment in 2015 would be below 0% at the end of 2021, while an investment in the control area would have generated a return of more than 43% over the same period.

This result does not only matter for households, who invest heavily in housing and would have suffered wealth losses, but also for firms and economic activity. In line with the collateral channel literature, the borrowing capacity of firms depends on the valuation of their real estate assets^64^. Manufacturing firms exposed to negative local house price shocks receive less credit from banks and their investment is reduced, decreasing the capital ratio, total factor productivity, and economic activity in the affected area^65^.

Figure 5 (middle) shows part of this transmission channel. The median mortgage principal amount in the control area has increased by more than 40% and it remains flat in Mar Menor, suggesting that banks have incorporated the lower value of housing in their lending decisions. Figure 5 (right) shows the number of transactions, which is a good proxy for economic activity^66^. These values were similar before the treatment in both areas. After the treatment, transactions almost double in the control area with respect to the treatment group, reflecting a differential increase in economic activity.

In addition to the effects on economic activity, shocks to real estate value have a direct impact on financial stability, as we mentioned in the main section. As a matter of fact, real estate variables are key indicators to measure the degree of financial stability in an economy^67^, mainly because of the role of real state wealth as collateral (wealth effect)^68^. Obviously, if this problem is limited to a very specific area, such as Mar Menor, it will have only a local effect. However, our results would still be indicative of the consequences of broader ecological deterioration, a risk that, indeed, could materialize more easily elsewhere in light of adverse developments related to climate change.

Answering the question on the wealth effect requires an estimation of the number of existing dwellings in the affected Mar Menor postcodes. According to cadastral statistics, the total number of dwellings in the Mar Menor area in 2015 was approximately 142,000. Given that the median size of dwellings is 72^2^ m and the median price per square meter was, in 2015, €1095, the total value of housing was €11,200 million. The missing returns of these dwellings relative to the control area is 43%, which implies a loss of wealth of €4800 million. For comparison purposes, an upper bound to the estimated gains in wealth from the change of dry-farming to crop-irrigation in the region, which is the main contributor to the fragility of this ecosystem is, in the period 2010–2019, of €443 million (for details of these calculations, see Supplement section 4).

## Discussion

We build a case-study by exploiting the fact that the area affected is delimited, as it comprises the houses along the Mar Menor coast. By comparing the evolution of house prices in this area with that of a control group of similar dwellings unaffected by the HAB, one can derive the HAB impact on house prices.

In our case, the control group is made up of dwellings located just a few kilometers north of Mar Menor, along the Mediterranean coast, which have not suffered the negative consequences of the HAB. In particular, these houses are located in South Alicante. We will show with granular data that this control area is not only adjacent to the Mar Menor, sharing therefore similar economic characteristics, but also that houses are of similar quality. In addition, the timeframe of the HAB is well-defined and was, as shown before, unanticipated by the population. All in all, we claim that this is an ideal setting for identifying the price effects of the HAB by means of a difference-in-differences (DD) approach. The DD method has been widely used in the academic literature to compare changes in outcomes over time between a population enrolled in a program (the treatment group) and a population that is not (the comparison group), particularly when the treatment is completely exogenous^69–71^.

Specifically, there was a treatment (the HAB) in the Mar Menor area after 2015. The purpose of the analysis is to check if housing prices in the treatment area after the treatment have evolved differently than prices in the control area. If this were the case, we could conclude that the HAB has had a negative effect on the evolution of house prices. We examine this possibility by means of a standard DD approach, detailed in the Methods section. This methodology tests whether price trends are the same before the treatment (parallel trend assumption) and whether they diverge after the treatment (treatment effect).

But, in addition to the treatment, other reasons might explain the diverging trends in the housing prices of the two areas. For example, if it is fashionable to buy big houses in recent years and the average house size is not the same in the two zones, we will observe distinct price trends unrelated to the HAB. In order to isolate HAB-related price effects, our DD specification incorporates a set of explanatory variables (control variables) which are mainly housing and transaction characteristics that are the usual determinants of house prices (see the Methods section).

**Figure 6:**
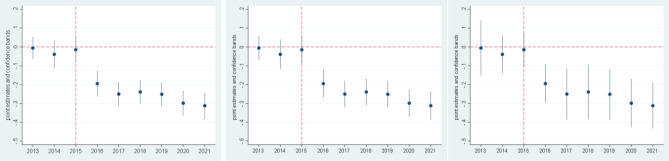
Differential effect of housing prices in Mar Menor vs control area. OLS estimation (left), heteroscedasticity consistent standard errors (middle), and cluster errors (right). The dots represent coefficient estimates of the differences in prices in every period between the treated area and the control area and can be interpreted as the differential growth rate of house prices in Mar Menor versus the control group The solid lines represent two standard errors bands.

However, even with these controls, it might be that somehow the houses in the control area and the treated area are different. There is one coefficient that measures this possibility, ${B}_{2013}$ which captures if the price per square meter in the treated area (Mar Menor) is different than in the control area, after taking into account the role of all the other explanatory variables. This is a good measure of the comparability of the housing market in the two zones. Our estimates of ${B}_{2013}$ are plotted in the first coefficient of Fig. 6. This figure presents the outcome of the estimation of the relevant coefficients of the DD equation, allowing for different variance specifications (for robustness). The first coefficient in the Figure corresponds to the difference in the price level (measured with the log of the price per square meter) between dwellings located in the treatment area and in the control area (in the base year, 2013). As can be seen in Fig. 6, in all specifications, we can accept the null hypothesis that ${B}_{2013}=0$. The areas are comparable because, controlling for different characteristics, house prices are not statistically different, and, therefore, the quality of dwellings is presumably the same.

The second key parameters to consider, are the ones that measures the parallel trend. The parallel trend assumption is critical to ensuring the internal validity of DD models. It requires that in the absence of treatment, the difference between the treatment and control group is constant over time. In our case, it is measured by the ${B}_{2014}$ and ${B}_{2015}$ coefficients. These parameters measure the differential behavior of the trend in housing prices in Mar Menor versus the control area up to 2015, before the HAB episode. It is the differential behavior because the common trend between these two areas is captured by the coefficients $\gamma$ (see methods section). Again, Fig. 6 shows very clearly that we can accept the null that ${B}_{2014}=0$ and ${B}_{2015}=0$. These coefficients are plotted in the second and third column of the figure. Given this evidence, we can conclude that, had there been no treatment (the HAB), housing prices in both areas would have evolved similarly over time.

Finally, after finding that the two areas are comparable and showing that, without the treatment, they would have had the same price evolution, the key parameters to analyze are those that show the differential evolution of housing prices after the treatment. These are measured by the coefficients ${B}_{2016}$ until ${B}_{2021}$.

The evolution of these coefficients is also plotted in Fig. 6. As can be seen in the figure, all the coefficients after ${B}_{2016}$ are negative and significant. This implies that there is a differential effect after the HAB episode, and that this differential effect is still present today.

With respect to the magnitude of the effect, given that the prices are specified in logs, the coefficients in the Figure can be interpreted as the differential growth rate of house prices in Mar Menor versus the control group, since the base year (2013). Therefore, controlling for housing heterogeneity, we can conclude that dwellings located in Mar Menor are sold at a price more than 30% lower in 2021 than the ones sold in the control area. This negative effect is similar to that documented in the previous section (43%), where we explicitly do not account for housing heterogeneity. Indeed, in some estimations and time periods, we would accept that DD estimates are not statistically different from the rough estimate calculated before. We also note that these effects are evident since the first year of treatment (2016), implying a significant loss for the average tenant of a house affected by the HAB, with all the economic consequences previously explained. All the coefficients of the estimation are displayed in Table A.5.1 of the Supplement.

In addition, section 6 of the supplementary material provides a statistical relation between the estimated values of the time coefficients in the treatment areas ${B}_{t}$ and the two measures of public perception proposed in the paper, Factiva and Twitter. In particular, we relate the coefficients ${B}_{t}$ for the sample 2014–2021 with the proportion of news containing “Mar Menor” from Factiva and the difference in the proportion of positive and negative tweets. The results show a solid statistical link between public perception of environmental degradation in Mar Menor and house price sensitivities. In particular, we find that an increase in the proportion of news about Mar Menor of one percentage point decreases the relative house prices in the Mar Menor area by 6 percentage points. For the same argument, a one percent increase in the proportion of net negative tweets implies a decrease of 0.4% points in the relative price in Mar Menor.

Finally, we add three additional robustness analyses. First, we restrict our sample to houses sold more than once in the sample to assure maximum homogeneity over time. Second, we change the control area adding all the coastal postal codes in Murcia Region not affected by the HAB. Finally, we include La Manga in the affected area. Results hold (see Methods and Supplement 5.2, 5.3 and 5.4 respectively).

In short, this paper provides a precise estimate of the economic impact of the environmental deterioration in the Mar Menor region and illustrates the importance of the physical risks of climate change for the valuation of assets, and their potential impact on financial stability.

## Methods

We use a total of 13,260 properties sold during the period 2013–2021 (October) in the control area and 8842 properties in the Mar Menor area. We use the DD estimator proposed extensively in the literature of treatment effects^69–71^. The estimated model is:

$${y}_{i}=\alpha +{B}_{2013}{Treat(Mar\,Menor)}_{i}+\sum\limits_{t=2014}^{t=2021}{\gamma }_{t}{Time}_{t,i}+\sum\limits_{t=2014}^{t=2021}{\beta }_{t}{Time}_{t,i}*{Treat(Mar\,Menor)}_{i}+{\delta }_{k}{{X}_{i,k}+\varepsilon }_{i}$$

where ${y}_{i}=$ Log of Price per square meter for house i, ${Treat(Mar Menor)}_{i}$= Dummy value 1 if House i belongs to Mar Menor, ${Time}_{t,i}$= Dummy value 1 if House i is sold in period t, ${X}_{i,k}$ Control variables

In our specification we include the following:

- ${X}_{i,1}$ Control for the type of house. Three dummies depending on the type, terraced house, isolated house, flat, flat with storage.
- ${X}_{i,2}$ House size: Four dummies, four cut points, 54, 64, 76, 94 sq meters.
- ${X}_{i,3}$ Construction status: Two dummies. Under construction, New, Second hand
- ${X}_{i,4}$ Buyer nationality: One dummy. Spanish or Foreign
- ${X}_{i,5}$ Seller nationality: One dummy. Spanish or Foreign
- ${X}_{i,6}$ Seller legal status: One dummy: Legal entity or individual

The model is estimated using Ordinary Least Squares techniques, with homoscedastic standard errors but, for robustness, we also allow for heteroscedasticity consistent standard errors^72^ and clustered standard errors that takes into account that different subgroups of houses might have their own source of uncertainty^73^. In our case, we allow for different variance by zip code and by housing size (5 buckets).

## Supplementary Information

Supplementary Information.

## Data Availability

Section: Public Perception of the HAB. Data on News: The data that support the findings of this study are available from FACTIVA (https://www.dowjones.com/professional/es/factiva/) but restrictions apply to the availability of these data, which were used under license for the current study, and so are not publicly available. Data are however available from the authors upon reasonable request and with permission of FACTIVA. Data on Twitter: The data and codes to extract data on Twitter are available here: https://gitfront.io/r/manolopy/51SqTCY8jckn/Twitter-Mar-Menor/. Explanations on the use of these codes can be found in the supplementary material of this paper. Section: Tale of two cities. The data are freely available here: https://www.idealista.com/sala-de-prensa/informes-precio-vivienda/. Data of the rest of the Sections. The data that support the rest of the findings of this study are available from “Estadística Registral Inmobiliaria del Colegio de Registradores de la Propiedad, Bienes Muebles y Mercantiles de España” but restrictions apply to the availability of these data, which were used under license for the current study, and so are not publicly available. Data are however available from the authors upon reasonable request and with permission of “Colegio de Registradores de la Propiedad, Bienes Muebles y Mercantiles de España”.
