# Supplementary material for: Impact of climate risk materialization and ecological deterioration on house prices in Mar Menor, Spain
Source: Sci Rep. 2023 Jul 21;13:11772. doi: 10.1038/s41598-023-39022-8 (PMC10362035; doi:10.1038/s41598-023-39022-8)
Supplement: Supplementary file 1 — Supplementary Information. [file 41598_2023_39022_MOESM1_ESM.docx]

Supplementary material for the paper:

Impact of Climate Risk Materialization and Ecological Deterioration on House Prices in Mar Menor, Spain

**Section 1: News about Mar Menor**

**Section 1.1. Structural Break tests with unknown breakpoint**

Calling the number of news (or the proportion of news containing Mar Menor over Murcia excluding sports) $y_{t}$, we estimate the model:

$$y_{t}=\alpha+\phi y_{t-1}+\varepsilon_{t}$$

and we test for the stability of the parameters $\alpha$ and $\phi$.

Andrews (1991, 1993), Andrews and Ploberger (1994), and Bai and Perron (1998), (2003a), (2003b) propose different methods to test for parameter instability when the breakpoint is unknown. Actually, the breakpoint is an additional parameter that needs to be estimated. The seminal proposed test by Andrews is simply the maximum of the F-statistics of the null hypothesis of no-break, assuming that the break point is in each of the possible dates of the sample (leaving at least 10% of the observations in each side of the subsamples). The proposed statistic is then:

$$Max F={Max}_{\tau_{1}<t<}\tau_{2} F(t)$$

Where $F(t)$ is the F statistic of the null hypothesis of no break for all t between $\tau_{1}$ and $\tau_{2}$

The results reject the null hypothesis of no break with a p-value of 0.02 and the estimated breakpoint, for the available sample, 2000-2021 is 2015. When we allow for multiple breakpoints using Bai and Perron specification, we find only one significant (at 5% confidence interval) break that also coincides with 2015. The dynamics of the period 2000-2015 are different from the dynamics of the period 2016-2021, which can be clearly seen in Figure 2, top graphs of the main text.

The results are comparable when we use the proportion of news with the word “Mar Menor” over the news, including “Murcia Region” after excluding sports news.

**Section 1.2. Change in topics about Mar Menor**


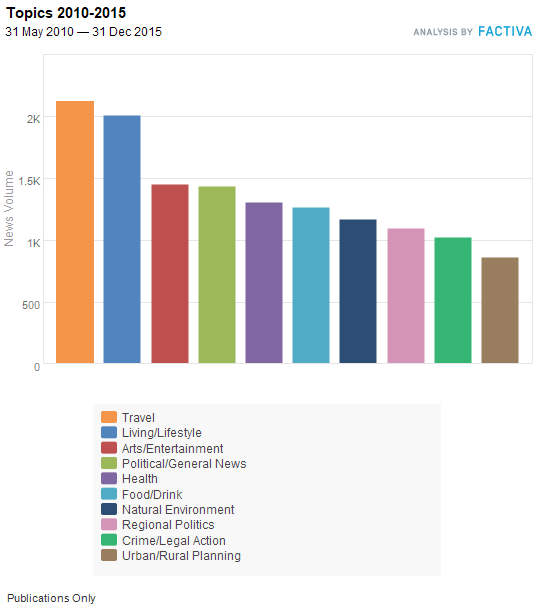

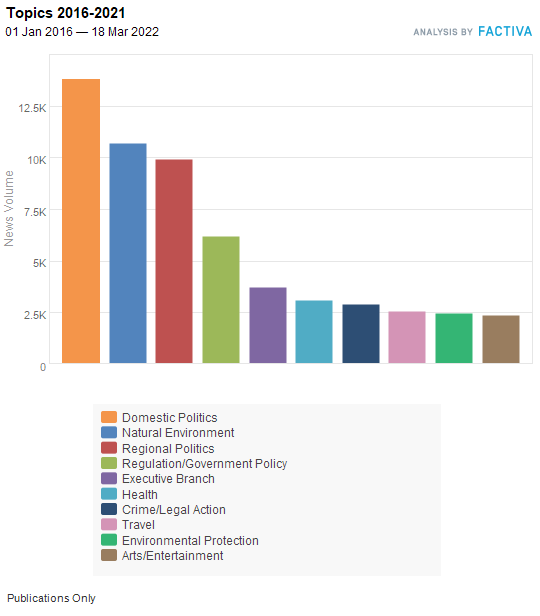


Figure A.1.2 News about Mar Menor by subject

In addition, as shown in Figure A.1.2, there is a substantial change in the subjects about Mar Menor. Before the break, we observe that news about travel, living/lifestyle, general news, arts/entertainment and food/drink are the more important ones, but after the break, politics, regulation, and the natural environment are the ones that make news. Therefore, not only the dynamics of the news are different, as shown in the previous section, but the news content is also very different.

**Section 2. Tweets about Mar Menor**

**Section 2.1 Extracting and classifying the tweets about Mar Menor**

All the codes can be found in:

<https://gitfront.io/r/manolopy/51SqTCY8jckn/Twitter-Mar-Menor/>

This section provides guidance to use the codes to create a database with tweets about Mar Menor and perform sentiment analysis to study how contamination affected the evolution of public opinion.

#First step: Generate database with mar_menor.py

This code includes the snscrape library, which requires to use the interpreter Python 3.8 to run. Previous versions cannot run this code. Posterior versions may lead to errors.

Snscrape is a library for web scraping from the history of social media programs such as Twitter, Facebook, Instagram, Reddit, etc. In this application, we focus on Twitter.

The code searches in Twitter for all the tweets containing the words “Mar Menor” that have been posted on a given day. This process is repeated in a loop every day from 1st January 2010 to 18th March 2022.

We can modify the parameters to change the maximum number of tweets we want per day, the starting date, and the ending date of the extraction. This code stores the date, the text and the username of every tweet filtered by the search. It creates one separated .xlsx by each year. We have three reasons to do this:

First, the code takes a long time to run. If there is any interruption in the execution, at least we conserve the database until the last completed year before the interruption, such that we only need to run this code for the remaining years instead of starting from the beginning.

Second, textual information weighs a lot. If we store everything in one dataframe within Python, the probability that it will run out of memory is very high.

Third, splitting the sample into different files has several computational advantages. We can perform parallel computing if we want to build or analyze the database faster, using many computers or threads simultaneously.

#Second step: Classify the tweets with Sentiment_analysis.py

This code uses as input the files generated in the previous step. It runs a loop that analyses in each iteration all the tweets about Mar Menor in a given year and generates a new .xlsx file with the sentiment data for that year, which contain the daily number of positive, negative, neutral and total tweets about Mar Menor.

First, it detects the language of the tweet with the library langdetect. Then we classify the subjective polarity of the tweets. This program can filter Spanish and English tweets.

To work with the Spanish tweets, we used the sentiment-spanish python library created by Hugo J. Bello (<https://pypi.org/project/sentiment-analysis-spanish)>. It uses convolutional neural networks to predict the probability of Spanish text being positive. We classified a tweet as positive, negative, or neutral if this probability is >0.6, <0.4 or between 0.4-0.6, respectively. This machine learning model was trained using over 800000 reviews of users of the pages el tenedor, decathlon, tripadvisor, filmaffinity and eBay. This model has a validation accuracy (accuracy over fresh data, not used for training) of 88%.

The algorithm for English tweets is VADER (Valence Aware Dictionary for Sentiment Reasoning), available in the NLTK Python Library. It is a lexicon (LEX) and rule-based model which has shown to outperform other rule-based and machine learning models previously used in the literature (Hutto and Gilbert, 2014). One of the VADER polarity score algorithm variables is called compound, and its sign tells us if the tweet is positive, negative, or neutral (compound >0, <0, =0 respectively).

Henriquez, Guzman, & Santamaria (2016) found that ML models work better for Spanish text, but Hutto and Gilbert (2014) concluded that LEX rule-based models are more efficient in classifying English textual data. These different results may be due to the differences in the training samples and linguistic resources available in both languages.

#Third step: We merge the sentiment data into a single .xlsx with merge.py

Once we have all the sentiment data, we merge all the sentiment data in a unique .xls

Then we group by week, adding up all the number of positive, negative, neutral and total tweets within each week. We do this to reduce the noise of the data because high-frequency data is very noisy. This way, the graphs are easier to interpret.

To group by week, we use pseudo_week_transformation.py. The first three pseudo weeks comprehend seven days, but the last one is from day 22 until the end of the month. We do this to make every month always have 4 pseudo weeks, which is standard in the economics literature; see, for example, Lewis et al. (2020). Otherwise, some months would have four weeks, and others would have five weeks, creating problems when using this data in mathematical or statistical models.

**Section 2.2 Testing for breaks in the tweets about Mar Menor**

We repeat the procedure of section 1.1 of this supplementary material, where the variable $y_{t}$ is the difference between the positive and negative tweets containing the word Mar Menor. We take a four week moving average of the data to avoid extremely volatile observations, and we estimate the model

$$y_{t}=\alpha+{\phi_{1}y}_{t-1}+{\phi_{2}y}_{t-2}+\ldots{\phi_{8}y}_{t-8}+{\phi_{9}y}_{t-9}+\varepsilon_{t}$$

Where we increase the number of lags with respect to the specification in 1.1 because we have higher frequency of data. We test for the joint stability of the parameters using the previously explained test of Andrews (1993) and the Bai and Perron test for multiple breaks. We find only one significant break, with a p-value of 0.01 in the second week of June 2016, coinciding with the reactivation of visitors in the area and their first-hand experience of visiting a deteriorated ecosystem.

**Section 3. A tale of two cities. Cointegration analysis.**

K time series, $y_{1,t}\ldots.y_{k,t}$ are integrated of order “d”(are non-stationary and need “d” differences to become stationary). If there is a linear combination across, then is integrated of order “d-1” these series are cointegrated.

Cointegration was introduced by the Nobel Prize Winners, Robert Engle and Clive Granger in their seminal work, Engle and Granger (1987). In economics, it usually refers to non-stationary series (usually random walks, d=1), which are strongly dominated in their signal by their stochastic trend, but a combination of these series is stationary (d=0). Engle and Granger (1987) also show that if K series are cointegrated, in the evolution of ${\Delta y}_{i,t} (i=1\ldots K)$, the residual of the cointegration relation (called the Error Correction Term) is statistically significant.

In the example that we are analyzing, we have two time series, $y_{1,t}$ the log prices in San Pedro (the area of San Pedro adjacent to Pilar de la Horadada, a district called Lo Pagan) and $y_{2,t}$the logs prices in Pilar de la Horadada.

Before the summer of 2015^[[1]](#footnote-1)^, (sample 2010.01-2015.06), applying the Johansen (1991) test, we find cointegration between these two price series (we reject no cointegration with a p-value of 0.03. Therefore, the growth rates of prices in San Pedro (${\Delta y}_{1,t})$ and growth rate of prices in Pilar de la Horadada (${\Delta y}_{2,t})$ are driven by its own past and a measure of the divergences between these two prices because their levels cannot be far away from each other. This divergence between these two prices is called error correction term. The estimated model is then:

$\left( \begin{matrix} {\Delta y}_{1,t} \\ {\Delta y}_{2,t} \end{matrix} \right)$ =$\left( \begin{matrix} c_{1} \\ c_{2} \end{matrix} \right)+\left( \begin{matrix} \emptyset_{1}(L) & \emptyset_{2}(L) \\ \emptyset_{3}(L) & \emptyset_{4}(L) \end{matrix} \right)\left( \begin{matrix} {\Delta y}_{1,t-1} \\ {\Delta y}_{2,t-1} \end{matrix} \right)+\left( \begin{matrix} d_{1} \\ d_{2} \end{matrix} \right)\left( y_{1,t}-c-\propto y_{2,t} \right)+\left( \begin{matrix} \varepsilon_{1} \\ \varepsilon_{2} \end{matrix} \right)$

$L$ represents the lag operator that indicates the number of lags of the variables included in the specification. $\left( y_{1,t}-c-\propto y_{2,t} \right)$ is the formal definition of the error correction term. The coefficients $d_{1}$and $d_{2}$ have the expected sign (negative for $d_{1}$and positive for $d_{2}$) which is indicative that, when prices in San Pedro are too high (low) with respect to prices in El Pilar de la Horadada $(\left( y_{1,t}-c-\propto y_{2,t} \right)$is very positive), they tend to go down (up), prices in El Pilar will have the tendency to go up (down). Also, as suggested by Engle and Granger (1987) at least one of them is significant. $d_{1}=-0.02 (0.03)$, $d_{2}=0.17 (0.07)$. Therefore, if prices in San Pedro drift positively (negatively) from prices in Pilar de la Horadada, prices in San Pedro will decrease (increase) in the following period and prices in Pilar de la Horadada will increase (decrease) to catch up with San Pedro prices. Associated to this specification and for L=6, we obtain the impulse response functions represented in figure A.3.1. Impulse response functions represent how one shock in one unit in the growth rate of the price in one location is transmitted to its own dynamics and in the dynamics of the other variable.

Figure A.3.1 Impulse response function. The graphs plot how a surprise of 1% in the growth rate of prices in each location is transmitted through the growth rates of the next 12 months to each of the two locations. The X axis contains the number of months after the shock, the Y axis is the variation in the growth rate of the prices in each location due to each particular shock. Figures are represented with 68% standard deviations bands.

As can be seen, shocks in San Pedro have a positive effect on housing prices in El Pilar de la Horadada (bottom left graph) and viceversa (upper right graph). These two series move together in the long run, therefore, shocks in the short run produce the same kind of movement in both series. Actually, it is remarkable how, after one year, shocks in El Pilar have a bigger effect on the growth rate of prices in San Pedro than shocks in San Pedro on itself. (two bottom graphs).

However, none of these results hold for the full sample (2010.01 2022.03). Johansen (1991) test accepts the null of no cointegration (p-value 0.17), and there is no significant transmission of shocks from one location to the other.

Nevertheless, one variable can explain the drift between these two price series. We introduce the difference between positive and negative tweets about Mar Menor (we transform the series to monthly frequency using the average of the weeks of a given month). The null of no cointegration using Johansen (1991) is rejected at 10%, p-value 0.06). It seems that there is a long term relation between $y_{1,t}$ the log prices in San Pedro, $y_{2,t}$the log prices in Pilar de la Horadada and $y_{3,t}$ the sentiment about San Pedro´s coastal area measured by the difference between positive and negative tweets. The estimated equation is now:

$\left( \begin{matrix} {\Delta y}_{1,t} \\ {\Delta y}_{2,t} \\ {\Delta y}_{3,t} \end{matrix} \right)$=$\left( \begin{matrix} c_{1} \\ c_{2} \\ c_{3} \end{matrix} \right)+\left( \begin{matrix} \emptyset_{1}(L) & \emptyset_{2}(L) & \emptyset_{5}(L) \\ \emptyset_{3}(L) & \emptyset_{4}(L) & \emptyset_{6}(L) \\ \emptyset_{7}(L) & \emptyset_{8}(L) & \emptyset_{9}(L) \end{matrix} \right)\left( \begin{matrix} {\Delta y}_{1,t-1} \\ {\Delta y}_{2,t-1} \\ {\Delta y}_{3,t-1} \end{matrix} \right)+\left( \begin{matrix} d_{1} \\ d_{2} \\ d_{3} \end{matrix} \right)\left( y_{1,t}-c-\alpha_{1}y_{2,t-}\alpha_{2}y_{3,t} \right)+\left( \begin{matrix} \varepsilon_{1} \\ \varepsilon_{2} \\ \varepsilon_{3} \end{matrix} \right)$

The results of the cointegration tests are confirmed because, as suggested by Engle and Granger (1987), at least one of the three parameters of interest is significant and with the appropriate sign, $d_{1}=-0.023 (0.010)$ and $d_{3}=0.126 (0.053)$. The coefficient $d_{1}$ reflects that prices in San Pedro separate positively (negatively) from what is expected from the sentiment and the prices in Pilar de la Horadada, they will go down (up) in the next period and $d_{3}$ implies that if the prices in San Pedro increases (decreases) with respect to its expected value, it will imply that positive (negative) sentiment will appear in the future to explain this price gap. The associated impulse response function for this specification is represented in figure A.3.2.

Figure A.3.2 Impulse response function. The graphs plot how a surprise of 1% in the growth rate of prices in each location (or an increase of 1 unit in the difference between the proportion of positive and negative shocks) is transmitted through the growth rates of the next 12 months to each of the two locations. The X axis contains the number of months after the shock, the Y axis is the variation in the growth rate of the prices in each location due to each particular shock. Figures are represented with 68% standard deviations bands.

As can be seen in the graphs, in addition to the transmission mechanism plotted before, we have to add that the sentiment variable affects the growth rate of prices in San Pedro. Good (bad) news about Mar Menor increases (decreases) the growth rate of prices in San Pedro (top left graph), as can be expected. Therefore, there are two transmission channels to the price in San Pedro, prices in El Pilar and sentiment variables.

**Section 4. Estimation of the number of houses and the wealth of changing from dry-farming to crop-irrigation.**

**Section 4.1 Number of houses.**

Cadastral statistics provide information about the number of houses built by municipality. Four municipalities have access to Mar Menor shores, the municipalities of San Javier, San Pedro del Pinatar, Los Alcazares and Cartagena, but the proportion of houses at the shore of Mar Menor in these municipalities is not the same. However, on the web page where owners put their houses up for sale (idealista.com), we have information on the number of houses for sale by district, with a map that allows us to calculate the proportion of houses for sale in the districts that are located on the sea shore with respect to the total number of houses for sale in the municipality. With this proportion, we can infer the proportion of total houses on the shore. Details of these calculations are shown in table A.4.1

Table A.4.1 shows the input for these calculations.

These numbers represent a lower bound to the number of houses because we are not considering the houses in the 20 Km strip of land called La Manga. Even though we exclude this area from the analysis because, as we said in the main text, it is only partially affected by the catastrophe (it has two shores, Mar Menor and Mediterranean), we show in section 5.4 of this supplementary material that the results are robust to include La Manga in the treated area. If we include this area, even assuming that only half of the properties are affected, the impact would be more than €6,250 million.

**Section 4.2 Wealth of changing from dry-farming to crop-irrigation.**

This section aims to calculate the wealth generated from dry-farming to crop-irrigation in the surrounding area of Mar Menor.

We calculate the gains from changing crops by measuring the increase in the value of the land changing from dry-farming to irrigation. Figure A.4.2 shows the evolution of the number of hectares in irrigation in the Murcia Region since the first period for which data are available. Trying to calculate an upper bound to the wealth accumulated by the farming sector in the surroundings of Mar Menor for changing the type of land, we consider the initial amount of land in irrigation as the one corresponding to the minimum of the time series (year 2010). We then assume that all the variation from the last period of the sample to the minimum is associated with an increase in irrigation land in Mar Menor (183,477 in 2021 minus 162,790 in 2010 =16,864 Ha). Therefore, we assume that the increase in irrigation of more than 16 thousand Ha only comes from the Mar Menor surroundings. Now we estimate the amount of wealth generated from this increase in irrigation. In order to do that, we compare (at 2021 prices) the value of these additional 16 thousand Ha as irrigation land with its value at dry-farming. The price of irrigation land is 37,126 Euros/Ha and the price of dry-farming is 4,690 Euros/Ha. The increase in price per Ha of changing the use of land is 32,436 Euros/Ha (prices of 2021 obtained from Centro Regional de Estadística de Murcia).

Therefore, the increase in wealth from changing the land use is 32,436 X 16,864= 547 million Euros, with is around ten times smaller than the destruction of wealth in the housing sector.

Figure A.4.2. Number of Hectares in irrigation in Murcia Region. Source: Encuesta sobre superficies y rendimientos de cultivos. Years 2004-2022

Additionally, we try more precise estimates by using a different source of information. On the one side, the Centro Regional de Estadística de Murcia provides information on changes from dry-farming to irrigated crops by municipalities. The data by municipalities start in 2013. We choose those municipalities that belong to the Mar Menor Area, San Pedro, Santiago de la Ribera, San Javier, Los Alcazares, Torre Pacheco, Cartagena and La Union. The maximum of irrigated land in this period is 36,993 Ha (year 2017); in the base year (2013), the irrigated land was 28,485 Ha. The change in the area is then 8,507 Ha. In the previous estimate, we consider the upper bound (we have a longer period, and we assume that all the changes in irrigated land are located in the Mar Menor area) of 16,864 Ha. The estimated gains from this precise estimate are half the gains estimated with the upper bound and, obviously, still order of magnitude smaller than the losses in housing.

Finally, thanks to the paper of Garcia Moreno et al (2018), we take into account the limitations of using official data for irrigated land. To calculate more appropriately the increase in irrigated land in the Mar Menor area, we now consider instead of the highest official number of irrigated land in the area (2017) of 37,000 Ha (in line with what we calculated using your previously suggested dataset), the additional 12,165 Ha that the authors calculate as illegal irrigation. Using as the base year the amount of irrigation in 1988 calculated by Carreño (2015) and that is quoted by Moreno et al (25,150 Ha), we have an increase in irrigated land from 1988 until 2017 of approximately 25,000. Evaluated at the price of irrigation land of 37,126 and the price of dry farming of 4,690, the increase in wealth from this change to irrigation land is 810 million Euros, still order of magnitude smaller than the losses to the value of housing due to the degradation of the Mar Menor ecosystem.

**Section 5. Difference in Difference**

**Section 5.1 Baseline model. Estimation coefficients**

We use the datasets of the Spanish Association of Registrars to carry out this analysis. We cover the universe of house transactions in this area. However, we exclude government-subsidized housing and houses purchased by legal entities since price determinants in these operations could be different. Our sample comprises 13,260 properties sold during the period 2013-2021 (October) in the control area and 8,842 properties in the Mar Menor area. We use the DD estimator proposed extensively in the literature of treatment effects. The estimated model is

$$y_{i}=\alpha+B_{2013}{Treat(Mar Menor)}_{i}+\sum_{t=2014}^{t=2021} \gamma_{t}{Time}_{t,i}+\sum_{t=2014}^{t=2021} \beta_{t}{Time}_{t,i}*{Treat(Mar Menor)}_{i}+\delta_{k}{X_{i,k}+\varepsilon}_{i}$$

Where:

- $y_{i}=$ Log of Price per square meter for the house i
- ${Treat(Mar Menor)}_{i}$= Dummy value 1 if House i belongs to Mar Menor
- ${Time}_{t,i}$= Dummy value 1 if House i is sold in period t
- $X_{i,k}$ Control variables

The model is estimated using OLS techniques, with homoscedastic standard errors, but, for robustness, we also allow for heteroscedasticity consistent standard errors (and clustered standard errors that take into account that different subgroups of houses might have their own source of uncertainty). In our case, we allow for different variance by zip code and housing size. We have then ten zip codes times five type of houses, creating 50 different variances.

In our specification we include the following:

- $X_{i,1}$ Control for the type of house. Three dummies depending on the type, terraced house, isolated house, flat, flat with storage
- $X_{i,2}$ House size: Four dummies, four cut points, 54, 64, 76, 94 sq meters.
- $X_{i,3}$ Construction status: Two dummies. Under construction, New, Second hand
- $X_{i,4}$Buyer nationality: One dummy. Spanish or Foreign
- $X_{i,5}$Seller nationality: One dummy. Spanish or Foreign
- $X_{i,6}$Seller legal status: One dummy: Legal entity or individual

To check for robustness, we also include zip dummies. The results are robust. All the estimated coefficients from the different specifications are displayed in Table A.5.1

**Section 5.2 Repeated houses. Estimation coefficients**

The estimation with repeated houses addresses the possibility that, even though houses sold in both areas are equal and the trends before the HAB were equal, the houses listed during the period 2016-2021 might be very different even when controlling for our set of explanatory variables. In order to address this possibility, we repeat the previous exercise, but we only use houses that are sold more than once in the period under study, just to make sure that the set of dwellings is completely homogeneous over time (e.g., before and after the HAB). The results for the variables of interest are displayed in Figure A.5.2, where we use the same format as the one used in Figure 6 of the main text, with the same three possible specifications for the variance.

Figure A.5.2 Differential effect of housing prices in Mar Menor vs control area. OLS estimation (left), heteroscedasticity consistent standard errors (middle), and cluster errors (right). Estimation with repeated houses.

The coefficient estimates are displayed in Table A.5.2. Obviously, in this case, all the coefficients related to dummies that do not change over time cannot be estimated.

**Section 5.3. Enlarging the control group.**

Murcia Region has other potential control areas that we could use in our analysis. South of the Mar Menor is clearly not comparable because a big city with an important port, Cartagena, is located, and, clearly, the price dynamics of such a different area are not comparable with Mar Menor. However, further south, in the same region, some other areas are potentially comparable (vacation houses, second residences, tourist destinations). We include these areas and we repeat the analysis developed in the main text. Our control area now has, instead of 13,260 houses, 18,456 units. Figure A.5.3.1 shows the map of the extended control and the treated area.


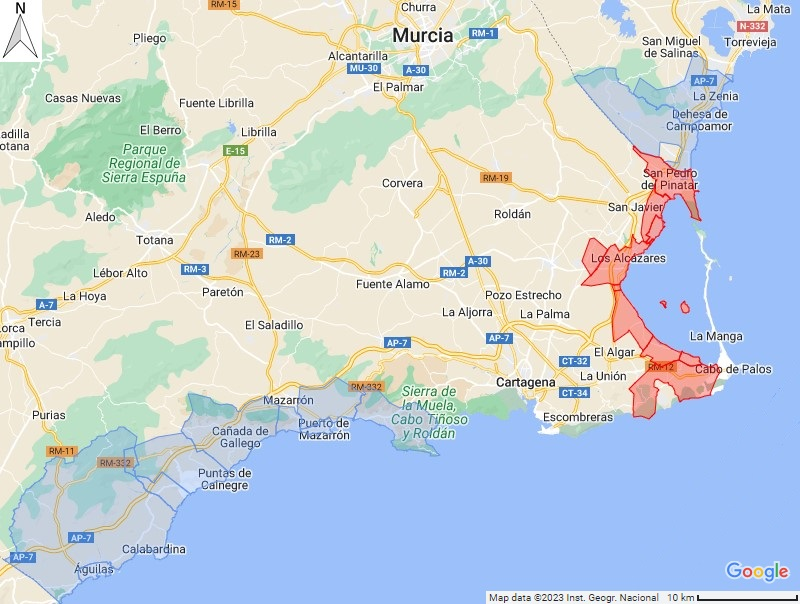


Figure A.5.3.1 Red area is the one affected by the ecological deterioration (Mar Menor). Blue area is the enlarged control group. Polygons were drawn with gmplot 1.4.1 (Python library: https://pypi.org/project/gmplot/) using data for ZIP coordinates from Goerlich (2022) that processes data from Centro Nacional de Información Geográfica (CNIG). Work derived from “CartoCiudad 2006-2021 CC-BY 4.0 scne.es” and “BDLJE CC-BY 4.0 ign.es”. Source of map: Google Maps.

The results of running our baseline specifications are rather comparable to those in the main body of the paper, and the relevant coefficients are plotted in Figure A.5.3.2. Contrary to the control area used in the main text, and even though the additional area apparently shares the characteristics of the Mar Menor, the new control area does not satisfy the strict requirements of the DD estimation. That is, even though the parallel trend assumption seems to marginally hold (we do not reject the null hypothesis that the coefficients in 2014 and 2015 are equal to 0), we clearly reject the homogeneity of the control and treated area in the base year (the coefficient of the treatment in the base year is positive and significant; prices in Mar Menor are higher than in the new control area). Even with this caveat, looking at the figure, it is clear that prices in Mar Menor decreased during the treatment period compared to prices in the new enlarged control area. Our results are robust, although a word of caution should be added with respect to the economic interpretation of our enlarged analysis.


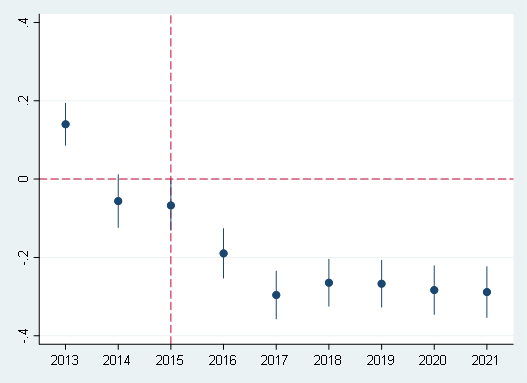

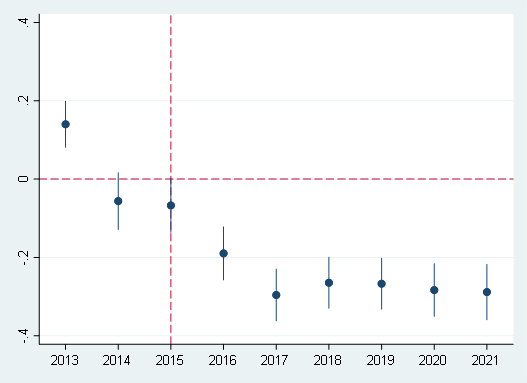

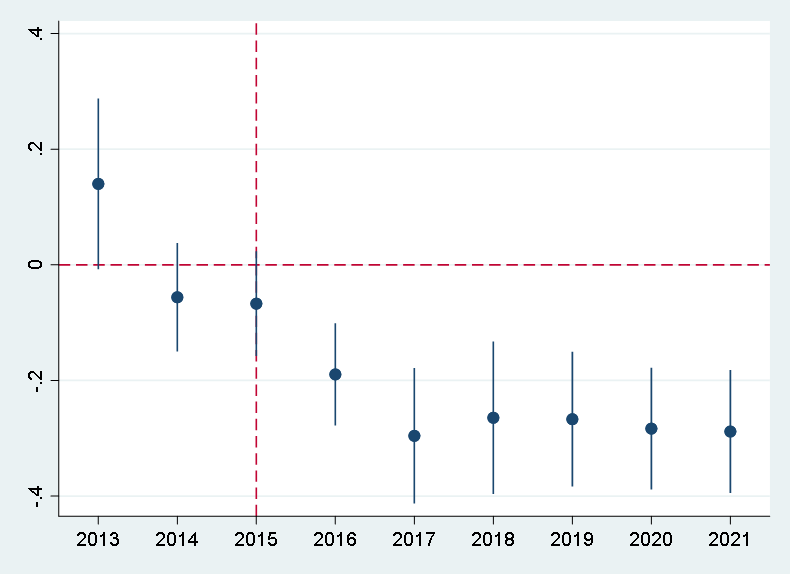


Figure A.5.3.2 Differential effect of housing prices in Mar Menor vs control area. OLS estimation (left), heteroscedasticity consistent standard errors (middle), and cluster errors (right). Estimation with enlarged control area.

**Section 5.4. Including La Manga Strip in the treated area.**

To understand the role of La Manga Strip in the robustness of our results, we propose different exercises. First, we include La Manga in the treated area (including it in the Mar Menor) and repeat the results of the paper. Left graph in Figure A.5.4 shows the results (for the heteroscedasticity consistent standard errors case). As can be seen, the results are remarkably similar to the ones presented in the paper. However, given that we add 3,165 houses to the estimation, the standard errors are smaller than the ones presented in the paper.

The middle graph presents the results of a second exercise. In this case, however, we consider as treated area only La Manga (and control area the one used in the paper, south of Alicante). As can be seen, the results are similar to the ones presented in the paper, although it seems that houses in La Manga are different (after including all the control variables) than the ones in the control area. This conclusion comes from the fact that we reject the null that the coefficient $B_{2013}=0$. Finally, in a third exercise, we compare houses in La Manga with the ones in the rest of Mar Menor (i.e. La Manga is the treated area and the rest of Mar Menor is the control area). The right graph plots the results of this analysis. As can be seen, houses in La Manga are different from houses in Mar Menor (we reject $B_{2013}=0$), and there is a marginal leakage effect (only significant in the last period of the sample) because it seems that houses in La Manga increase their price with respect to the rest of Mar Menor.

Therefore, to conclude the analysis, even though houses in La Manga are different than the rest of the houses in Mar Menor, they are equally affected by the episodes of HAB and the dynamics of their prices are the same than the dynamics of the rest of the Mar Menor area. With these results in mind, it is clear that the results of the main text represent a lower bound to the impact on housing wealth in the Mar Menor area.


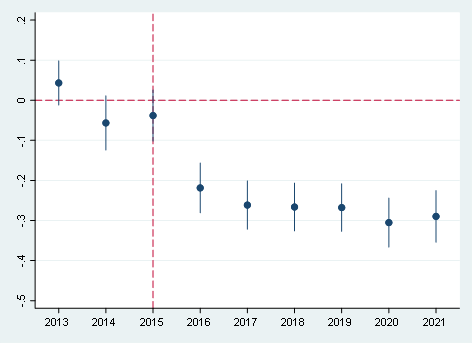

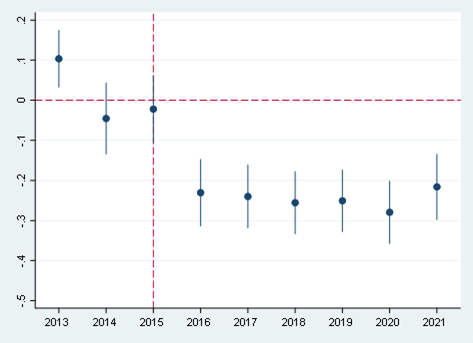

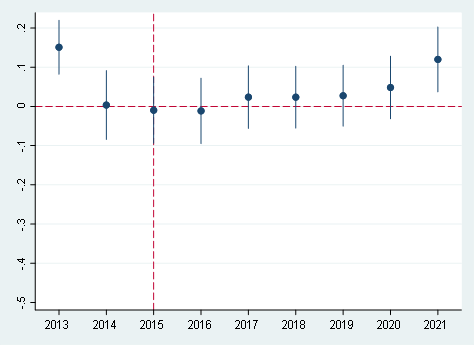


Figure A.5.4. Differential effect of housing prices in Mar Menor including La Manga strip vs control area (left). Differential effect of housing prices in La Manga versus the control area (middle). Differential effect of housing prices in La Manga vs rest of the Mar Menor (right). All the graphs present heteroscedasticity consistent standard error bands.

**Section 6. Relating the coefficients of the Difference in Difference with news and sentiment**

In this section we relate the values of the time coefficients related to the treatment areas ${(\beta}_{t})$ and the two measures of public perception proposed in the paper, Factiva and Twitter. In particular, we relate the coefficients ${(\beta}_{t})$ for the sample 2014-2021 with the proportion of news containing “Mar Menor” from Factiva and the difference in the proportion of positive and negative tweets. Even though the number of observations is very small, the results show a solid statistical link between public perception of environmental degradation in Mar Menor and house price sensitivities. Specifically, we run two standard linear regressions, where the dependent variable is ${(\beta}_{t})$ in both cases, and the independent variables ${(X}_{t})$ are either the Factiva or the tweets variable.

The regression proposed is:

$$\beta_{t}=a+bX_{t}+\varepsilon_{t}$$

The estimation of the parameter $b$ is, for the case of the Factiva variable (proportion of news containing Mar Menor with respect to news about Murcia), -0.062 (p-value 0.03). The interpretation of the coefficient is that an increase in the proportion of news about Mar Menor of one percentage point decreases the house prices in the Mar Menor area by 6 percentage points when compared to house prices in the control group. The $R^{2}$ of this regression is 0.79, which implies that most of the variance of $\beta_{t}$ can be explained by the model.

In the case of the number of negative tweets the results are similar. The estimation of the parameter $b$ is, in this case, 0.004 (p-value 0.009), which implies that an increase of 1% in the proportion of net negative tweets (negative – positive) implies a decrease of 0.4 percentage points in the price of housing in Mar Menor, when compared to house prices in the control group.

References of Section 1

Andrews D., “Heteroskedasticity and Autocorrelation Consistent Covariance Matrix Estimation,” Econometrica 59(3) (1991), 817–58.

Andrews, D. W. (1993). Tests for parameter instability and structural change with unknown change point. Econometrica: Journal of the Econometric Society, 821-856.

Andrews, D. W. (2003). End‐of‐sample instability tests. Econometrica, 71(6), 1661-1694.

Andrews, D. W., & Ploberger, W. (1994). Optimal tests when a nuisance parameter is present only under the alternative. Econometrica: Journal of the Econometric Society, 1383-1414.

Bai, J., & Perron, P. (2003). Computation and analysis of multiple structural change models. Journal of applied econometrics, 18(1), 1-22.

Bai, J., & Perron, P. (2003). Critical values for multiple structural change tests. The Econometrics Journal, 6(1), 72-78. References of Section 2

Henriquez Miranda, C. N., Guzman, J., & Santamaria, R. (2016). A review of Sentiment Analysis in Spanish. TECCIENCIA, 12(22), 40–47. Retrieved from <https://revistas.ecci.edu.co/index.php/TECCIENCIA/article/view/320>

Hutto, C., & Gilbert, E. (2014, May). Vader: A parsimonious rule-based model for sentiment analysis of social media text. In Proceedings of the international AAAI conference on web and social media (Vol. 8, No. 1, pp. 216-225).

Lewis, D. J., Mertens, K., Stock, J. H., & Trivedi, M. (2022). Measuring real activity using a weekly economic index. Journal of Applied Econometrics, 37(4), 667-687.

References of Section 3

Engle, R. and Clive Granger (1987). Co-integration and Error correction: Representation, estimation and testing. Econometrica, Vol 55 No 2 March 1987, pages 251-276

Johansen, Søren (1991). "Estimation and Hypothesis Testing of Cointegration Vectors in Gaussian Vector Autoregressive Models". Econometrica. 59 (6): 1551–1580

References of Section 4

Encuesta sobre Superficies y Rendimientos de Cultivos en España, ESYRCE, (2021). Gobierno de España. Ministerio de Agricultura, Pesca y Alimentación. Subsecretaría de Agricultura, Pesca y Alimentación, Subdirección General de Análisis Coordinación y Estadística.

Carreño, M.F. 2015. Seguimiento de los cambios de usos y su influencia en las comunidades y hábitats naturales en la cuenca del Mar Menor, 1988-2009, con el uso de SIG y Teledetección. Tesis Doctoral. Universidad de Murcia

Garcia Moreno, Pedro, Antonio D. Ibarra Marinas and Jorge M. Sánchez Balibrea (2018). La Burbuja del Regadío: El caso del Mar Menor. 1977-2017. ANSE y WWF España.

References of Section 5

Goerlich, F.J. (2022). «Elaboración de un mapa de códigos postales de España con recursos libres. Cómo evitar pagar por información de referencia». Working Papers Ivie n.º 2022-3. València: Ivie. Available at: <http://doi.org/10.12842/WPIVIE_0322>

1. We choose Summer 2015 because the summer of 2015 coincides with the first phytoplankton proliferation episode documented and, given the proximity of these towns, prices seems to react a few months earlier than in the whole treatment area. [↑](#footnote-ref-1)
